# Bacterial Heat Shock Protein GroEL (Hsp64) Exerts Immunoregulatory Effects on T Cells by Utilizing Apoptosis

**DOI:** 10.1371/journal.pone.0164380

**Published:** 2016-10-13

**Authors:** Ayten Nalbant, Melis Kant

**Affiliations:** 1 Department of Molecular Biology and Genetics, Izmir Institute of Technology, Izmir, Turkey; 2 Department of Medical Biochemistry, Dokuz Eylul University, Izmir, Turkey; Universidad de Palermo, UNITED STATES

## Abstract

*Aggregatibacter actinomycetemcomitans* (Aa) expresses a 64-kDa GroEL protein belonging to the heat shock family of proteins. This protein has been shown to influence human host cells, but the apoptotic capacity of the GroEL protein regarding T cells is not yet known. The purpose of this study was to investigate the ability of *A*. *actinomycetemcomitans* GroEL (AaGroEL) protein to induce human peripheral blood T-cell apoptosis. Endogenous, purified AaGroEL protein was used as an antigen. In AaGroEL-treated T cells, the data indicated that phosphatidylserine exposure, an early apoptotic event, was dose- and time-dependent. The AaGroEL-treated T cells were also positive for active caspase-3 in a dose-dependent manner. The rate of AaGroEL-induced apoptosis was suppressed by the addition of the general caspase inhibitor Z-VAD-FMK. Furthermore, cleaved caspase-8 bands (40/36 kDa and 23 kDa) were identified in cells responding to AaGroEL. DNA fragmentation was also detected in the AaGroEL-treated T cells. Overall, we demonstrated that the endogenous GroEL from *A*. *actinomycetemcomitans* has the capacity to induce T-cell apoptosis.

## Introduction

Apoptosis can be activated by a wide range of stimuli, including bacterial antigens [1]. One class of bacterial antigens is the heat shock protein 60 (Hsp60) family, which includes bacterial GroEL proteins. In addition to having a well-known role in protein folding, bacterial GroEL proteins are highly conserved and immunogenic [2, 3]. One type of GroEL-expressing bacteria is the periodontal pathogen *Aggregatibacter actinomycetemcomitans* (Aa), a gram-negative, facultative, non-motile bacterium that lives in the oral cavity [4, 5]. This pathogen is often associated with periodontal diseases [6, 7], which can affect periodontal tissues such as the gingival tissue and the alveolar bone. *A*. *actinomycetemcomitans* has also been implicated in several non-oral infections [8].

The stable presence of *A*. *actinomycetemcomitans* in periodontal tissues suggests that the attenuation of an exacerbated host response may be driven by pathogenic virulence factors to avoid the clearance of the pathogen by the immune system. Previously, it was shown that *A*. *actinomycetemcomitans* GroEL (AaGroEL) is mitogenic for cultured epithelial cells at low concentrations but cytotoxic at higher concentrations or upon prolonged exposure [9, 10]. Knockout mutants of *A*. *actinomycetemcomitans* with deletions of *ltxA* [11] and all 3 *cdt* genes [11] retained significant cytotoxicity. This observation suggests the existence of other cytotoxic molecules, distinct from cytolethal distending toxin (*CDT*) and leukotoxin (*LTX*), as important virulence factors of this bacterium [11]. However, these studies [9, 10, 11] did not measure the apoptotic ability of the GroEL protein from *A*. *actinomycetemcomitans*. Therefore, it is important to unravel the potential apoptotic properties of endogenous purified AaGroEL. Furthermore, no studies have yet investigated the apoptotic effect of AaGroEL on primary human T cells.

Apoptosis is characterized by a variety of molecular and morphological changes. One of the earliest events in the apoptotic cascade is a change in the plasma membrane. Phosphatidylserine (PS) is translocated from the inner to the outer leaflet of the plasma membrane, while maintaining the integrity of the membrane. Only at the culmination of apoptosis does the loss of plasma membrane integrity occur [12]. Another molecular hallmark of apoptosis is the activation of caspases, which are inactive enzymes that must become active during apoptosis. For example, caspase-8, a cytosolic protein that has homology to the CD95/Fas-associated death domain (FADD), is involved in the apoptotic process. The N-terminal region of caspase-8 contains an amino acid sequence, termed the death domain, which facilitates a direct caspase-8-FADD interaction. It is important to show that caspase-8 is activated in receptor-mediated apoptosis [13]. One of the effector caspases that becomes active during late apoptosis is caspase-3. Active caspase-3 initiates a series of events that ultimately kills cells [13]. DNA fragmentation is also a hallmark of apoptosis.

In this study, we used purified endogenous AaGroEL protein as a model antigen to study bacterial GroEL (Hsp64)-mediated apoptosis of primary human T cells. For this purpose, human peripheral blood mononuclear cells (PBMCs) were cultured with AaGroEL, and the apoptotic characteristics of T cells were measured. Our data suggest that endogenous AaGroEL induces T-cell apoptosis as determined by cell size, plasma membrane changes, caspase-3 and caspase-8 activation and DNA fragmentation.

## Materials and Methods

### Human subjects

Ethics approval for this study was obtained from the Noninvasive Ethics Committee of Dokuz Eylül University, İzmir, Turkey. All the donors were asked to sign an informed consent form. All the blood donors were periodontally and systemically healthy adult volunteers who were non-smokers under the age of 50. A total of 35 donors participated in this study. Venous blood was drawn from the volunteers by health professionals at the Izmir Institute of Technology Health Service. PBMCs were isolated using the Ficoll-Hypaque density gradient centrifugation method [14].

### Preparation of the GroEL antigen

The *A*. *actinomycetemcomitans* (29522) type strain was obtained from American Type Cell Culture (ATCC) (Rockville, MD). *A*. *actinomycetemcomitans* was grown as previously described [15]. To induce heat shock protein expression, bacterial cultures were incubated at 43°C for 1 h in a water bath [15]. Endogenously expressed GroEL protein was purified from an *A*. *actinomycetemcomitans* cell extract (AaCE) by adenosine 5’-triphosphate (ATP) affinity chromatography and electroeluted from an SDS-PAGE gel [15]. Briefly, ATP-agarose (AppliChem, Darmstadt, Germany) and a gravity column were used. The collected ATP affinity chromatography fractions were analyzed by SDS-PAGE. After staining with CuCl_2_, the bands corresponding to the 64-kDa GroEL protein were excised, destained and electroeluted. The protein concentration of purified samples was determined using the Bradford protein assay. Samples were stored at -20°C and used as an antigen.

Previously produced recombinant-AaGroEL protein (rAaGroEL, 200 ng) was used as a positive control for western blot experiments [16]. To verify the purified GroEL protein of *A*. *actinomycetemcomitans*, western blot analysis was performed with a primary mouse antibody directed against the *E*. *coli* recombinant GroEL (*E*. *coli* rGroEL) protein (1:5000) and an HRP-conjugated anti-mouse secondary antibody (1:20,000) (StressGen Biotechnologies, San Diego, CA) [16]. The relevant protein bands were also confirmed independently via LC-ESI-MS by Proteome Factory (Berlin, Germany). The lipopolysaccharide (LPS) concentration in purified AaGroEL samples was measured using Limulus amebocyte lysate (LAL) Chromogenic Endpoint assay kit (Hycult Biotechnology, Uden, Netherlands). Detoxi-Gel Endotoxin Removal Gel (Thermo, Fisher Scientific Inc., Waltham, Massachusetts) was used to remove LPS from the purified samples according to the manufacturer’s instructions.

### Cell cultures and stimulants

PBMCs were cultured from 0–96 has described previously [11]. RPMI alone was used as a negative control, while camptothecin (CPT, 4 μM) (Sigma-Aldrich, St. Louis, MO) was used as a positive control of the apoptosis. Purified, electroeluted-endogenous- AaGroEL protein (1, 50, 100, 250, 500 and 1000 ng/mL) was used as antigen. Commercially available *E*. *coli* recombinant GroEL protein (*E*. *coli* r GroEL) (StressGen Biotechnologies, San Diego, CA) and bovine serum albumin (BSA) (Sigma-Aldrich, St. Louis, MO) were purified by electroelution using the same method as for the AaGroEL protein and were used as a control of the purification process.

### Detection of plasma membrane changes

Cells were washed and labeled first with anti-CD3 antibody for cell-surface staining of T cells. The cells were then concurrently labeled with Annexin V (1:5) and 7-amino-actinomycin D (7AAD) (1:20) in the presence of a Ca^2+^-binding buffer (Becton Dickinson, Franklin Lakes, New Jersey) for 30 min and analyzed by flow cytometry within an hour. The cells were gated on the CD3 molecule for flow cytometric analysis.

Annexin V is a protein that has a high affinity for PS in the presence of Ca^2+^. During apoptosis, PS from the inner face of the plasma membrane translocates to the cell-surface face. The exposed PS can be detected by staining with fluorophore-conjugated Annexin V. 7AAD is a DNA-binding probe that is efficiently excluded by intact cells and is useful for discriminating dead cells during flow cytometric analyses. Annexin V staining is typically used in conjunction with the viability dye 7AAD to identify early apoptotic cells. Cells positive only for Annexin V (Annexin V+ 7AAD- cells) are early apoptotic, while double-positive cells (Annexin V+ 7AAD+ cells) are late apoptotic, and cells positive only for 7AAD (Annexin V- 7AAD+ cells) are dead cells.

### Detection of active caspase-3

Cells were first treated with an anti-CD3 monoclonal antibody to label T cells. Then, the cells were fixed and permeabilized, and an antibody specific for active caspase-3 (1:5) (Becton Dickinson, Franklin Lakes, New Jersey) was added to the cells for 20 min at 4°C. The washed cells were analyzed by flow cytometry. To inhibit the activity of caspases, PBMCs were pretreated with 50 μM Z-VAD-FMK (Becton Dickinson, Franklin Lakes, New Jersey), a general caspase inhibitor (CI), for 1 h. After the incubation, the cells were washed and cultured with AaGroEL for 72 h. The cells were then labeled with anti-CD3 antibody (1:5), Annexin V (1:5) and 7AAD (1:20) and analyzed by flow cytometry.

### Detection of caspase-8 by western blot analysis

Cells were washed, pelleted and lysed with RIPA lysis buffer (Cell Signaling Technology, Leiden, Netherlands) and a protease inhibitor cocktail (1:100). The cell lysate was separated on a 10% SDS-PAGE gel. The bands were transferred to a PVDF membrane and used for the western blot analysis. An anti-caspase-8 primary monoclonal antibody (1:5000) and HRP-conjugated anti-mouse IgG secondary antibody (1:20,000) (Cell Signaling Technology, Leiden, Netherlands) were used to detect caspase-8. The membrane was treated with a substrate solution for chemiluminescence (Thermo Scientific, Waltham, Massachusetts), and the bands were visualized with a VERSADOC 4000MP (Bio-Rad, Hercules, CA) instrument.

### Detection of DNA fragmentation by fluorescence microscopy

DNA fragmentation was detected using the MEBSTAIN Apoptosis Kit Direct (Immunotech, Marseille, France). This kit is based on the TUNEL [terminal deoxynucleotidyl transferase (TdT) dUTP nick-end labeling] method. DNA fragmentation generates 3’-OH DNA ends, which are then detected by labeling the terminal ends of the nucleic acids; i.e., the nick end is labeled with fluorescein (FITC)-dUTP mediated by TdT. Briefly, AaGroEL-treated or untreated negative control cells were washed with 1 mL of 1x PBS with 0.2% BSA and fixed with 4% paraformaldehyde buffer for 30 min at 4°C. After fixation, the cells were washed, and the pellet was permeabilized with 100 μL of 70% ethanol for 30 min at -20°C. The cells were then washed with 1 mL of 1x PBS containing 0.2% BSA, and the ethanol was discarded. The fixed and permeabilized cells were re-suspended in the DNA-labeling solution, TdT and FITC-conjugated dUTP, and incubated for 1 h at 37°C. After incubation, the cells were washed with 1x PBS containing 0.2% BSA and analyzed by fluorescence microscopy (Magnification x20) (Olympus, Tokyo, Japan).

### Statistical analysis

Samples were assayed with a FACSArray instrument (Becton Dickinson, Franklin Lakes, New Jersey), and the data were analyzed using the FACSArray system software and FlowJo software. The flow cytometry data were exported to MS Office Excel for further analysis. All the samples were analyzed in triplicate and were compared to the negative control using Student’s t test. A two-tailed Student’s t test was used for statistical analysis, and p<0.05 was accepted as statistically significant.

## Results

### Verification of purified GroEL protein by western blot analysis

**Fig 1:**
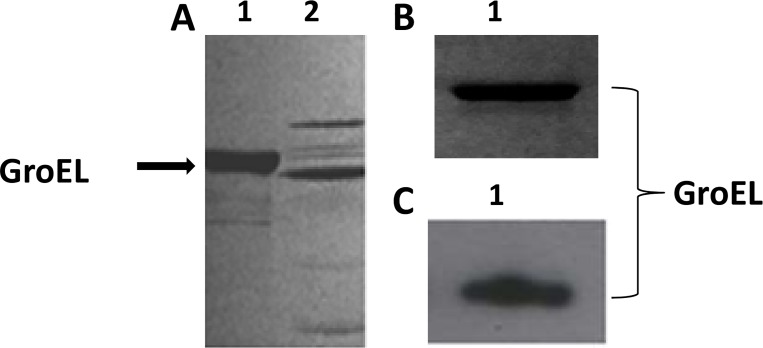
Purified GroEL protein confirmed by western blot analysis. (A) Purification of AaGroEL by ATP affinity chromatography. A cell extract of *Aggregatibacter actinomycetemcomitans* was loaded onto an adenosine 5’-triphosphate (ATP) agarose gel and fractions were collected with a 5 mM ATP solution. The ATP fractions were loaded onto an 8% SDS-PAGE gel. Lane 1, recombinant AaGroEL and Lane 2, ATP fraction. (B) Purification of AaGroEL by electroelution. The bands corresponding to the 64-kDa AaGroEL protein were excised from the SDS gel, and the slices were destained and electroeluted for 4 h. Purified AaGroEL was analyzed by SDS-PAGE, and the band was stained with Coomassie brilliant blue R-250. Lane 1, recombinant AaGroEL (200 ng) as a control [16] and Lane 2, electroeluted, purified AaGroEL (200 ng). (C) Native endogenous purified AaGroEL protein was confirmed with western blot analysis using an anti-*E*. *coli* GroEL antibody. Lane 1, rAaGroEL (200 ng) and Lane 2, endogenous AaGroEL (200 ng).

GroEL is a molecular chaperone that has an affinity for ATP. Therefore, ATP affinity chromatography was used to purify the GroEL protein from the *A*. *actinomycetemcomitans* cellular extract. SDS-PAGE analysis of the affinity-purified proteins revealed the presence of several proteins in the sample, indicating that the ATP chromatography fractions were not sufficiently pure (Fig 1A, lane 2). Therefore, the 64-kDa GroEL protein was electroeluted from the SDS-PAGE gel (Fig 1B), and western blotting was performed to verify the GroEL protein (Fig 1C, lane 2). Furthermore, the GroEL protein was also confirmed by mass spectrophotometry. The amino acid coverage was 39%, suggesting that the sequenced peptides belonged to GroEL (data not shown). The electroeluted AaGroEL was used in all succeeding experiments.

### GroEL induces T-cell apoptosis in a dose- and time-dependent manner

**Fig 2:**
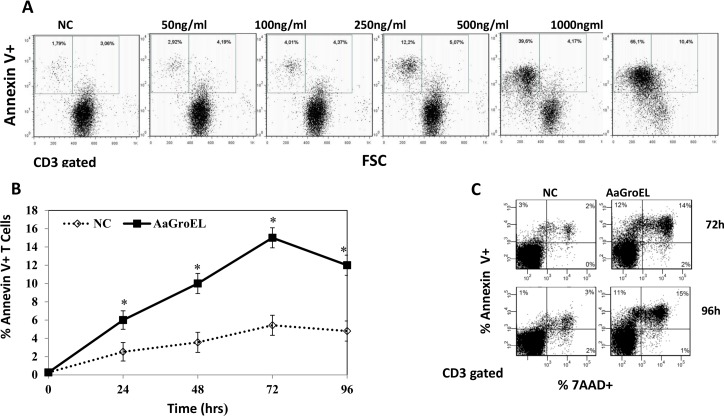
GroEL induces T-cell apoptosis as assessed by exposure of phosphatidylserine. (A) Dose-response of AaGroEL. PBMCs were cultured for 48 h with various AaGroEL concentrations (1–1000 ng/mL). CD3-gated dot plots of Annexin V labeling versus forward light scatter (FSC) are shown. The windows demarcate two subsets of Annexin V-positive T lymphocytes: the left window displays small-sized lymphocytes, and the right window displays medium-sized lymphocytes. (B) Time kinetics of AaGroEL. PBMCs were cultured with AaGroEL (250 ng) for various intervals (0–96 h). RPMI was used as a negative control and camptothecin (CPT, 4 μM) was used as a positive control. The cultured cells were labeled with anti-CD3 antibody, Annexin V and 7AAD followed by flow cytometric analysis. Error bars represent the standard deviation, and * indicates p<0.05. The data are representative of 6 experiments. (C) Representative flow cytometric profile of apoptotic T cells at 72 h and 96 h. Cells were gated for T lymphocytes based on CD3 expression. The proportion of Annexin V-positive T cells (pre-apoptotic) is indicated in the upper left quadrant of each panel, and Annexin V and 7AAD double-positive (apoptotic/necrotic) T cells are indicated in the upper right quadrant of each panel. The presented data are from one of six experiments.

The presence of inner-membrane PS on the outer membrane of a cell is one of the earliest events in apoptosis. This process can be monitored using Annexin V, a calcium-dependent protein that binds to negatively charged PS [12]. Additionally, 7AAD is a DNA-intercalating dye that enters cells when the integrity of the plasma membrane is lost. Therefore, staining with Annexin V in conjunction with 7AAD allows the identification of early (Annexin V-positive, 7AAD-negative) and late (Annexin V-positive, 7AAD-positive) apoptotic cells. To investigate changes in the plasma membrane phospholipid (PS) associated with apoptosis, PBMCs were cultured with different concentrations (1–1000 ng/mL) of AaGroEL for 48 h. During the flow cytometric analysis, cells were gated on CD3. Dot plots of Annexin V labeling versus forward light scatter (FSC), a measure of cell size, were used to monitor apoptotic cells. This analysis illustrated that after AaGroEL stimulation, cells were small in size and Annexin V-positive. The population of medium- to small-sized Annexin V-positive cells increased from 5% to 76% with 1000 ng/mL AaGroEL. The fraction of early apoptotic cells (only Annexin V positive) in AaGroEL-stimulated T cells was 7% at 50 ng/mL, 8% at 100 ng/mL, 17% at 250 ng/mL, 45% at 500 ng/mL and 76% at 1000 ng/mL (Fig 2A). These results clearly show that AaGroEL induces T-cell apoptosis in a dose-dependent manner (Fig 2A). A concentration of 500 ng/mL of GroEL was required to induce apoptosis in 50% of the T cells at 48 h (LD50). However, 250 ng/mL of protein was sufficient to observe a statistically significant difference (approximately 4-fold difference compared to the negative control, p<0.05, Fig 2A), and this dose was chosen for use in all subsequent experiments. Next, to study the kinetics of the plasma membrane changes in T cells, PBMCs were cultured with AaGroEL (250 ng/mL) from 0 to 96 hours. The percentage of only Annexin V-positive, early-apoptotic cells, was 6% at 24 h, 10% at 48 h, 15% at 72 h and decreased to 12% at 96 h (Fig 2B). There was a statistically significant difference (approximately 3-fold difference) between the AaGroEL-treated cells and the negative control at 72 h, p<0.05 (Fig 2B). These data indicated that endogenous AaGroEL induced apoptosis in a time- and dose-dependent manner (Fig 2A and 2C).

### GroEL induces T-cell apoptosis by activating caspase-3 and caspase-8

**Fig 3:**
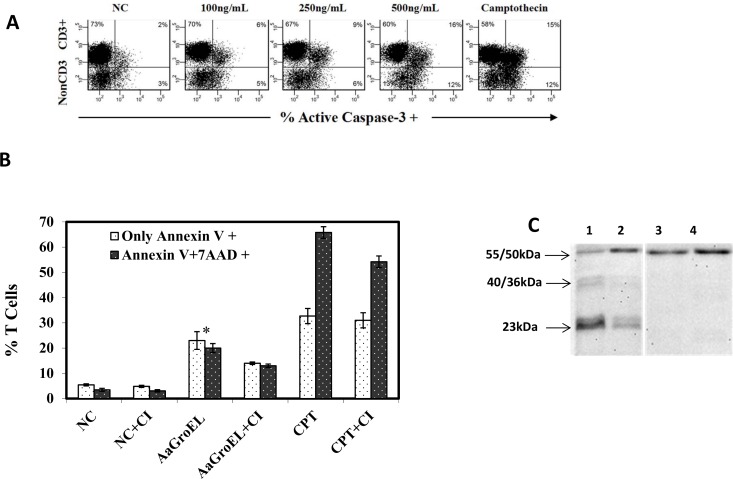
GroEL induces T cell apoptosis by activating caspases-3 and -8. (A) Caspase-3 activation in T cells. PBMCs were cultured for 72 h with various AaGroEL doses. RPMI alone (NC) and camptothecin (CPT, 4 μM) were used as negative and positive controls, respectively. Cells were first labeled with anti-CD3 antibody. The cells were then fixed and permeabilized, and anti-active caspase-3 antibody was added, followed by analysis using flow cytometry. The representative flow data indicate the active-caspase-3 level (%) of T cells (CD3+) and non-CD3+ cells. (B) Caspase inhibition assay. PBMCs were incubated with a general caspase inhibitor (CI; Z-VAD-FMK) for 1 h at 37°C before antigenic stimulation. After this incubation, the PBMCs were cultured with AaGroEL (250 ng/mL) and camptothecin (CPT, 4 μM) for 72 h. At the end of the culture, the cells were labeled with anti-CD3 antibody, Annexin V and 7AAD and analyzed by flow cytometry. Error bars represent the standard deviation, and * indicates p<0.05. The data are representative of three experiments with different donors. (C) Caspase-8 activation. PBMCs were cultured with AaGroEL (250 ng/ml) protein for 72 h and 48 h (lanes 1 and 2, respectively). RPMI was used as a negative control (lanes 3 and 4). The cells were probed with anti-human caspase-8 antibody and analyzed by western blotting. The 55/50 kDa procaspase-8 was detected in all the samples (lanes 1 to 4). The cleaved 40/36-kDa (doublet) and 23-kDa caspase-8 bands were seen only in AaGroEL-stimulated cells at 72 h and 48 h, respectively.

To determine whether caspase-3 activation is involved in AaGroEL-mediated T-cell apoptosis, we measured the active caspase-3 levels in cells cultured with different concentrations of AaGroEL for 72 h. The data revealed that the active caspase-3 level was 6% at 100 ng/mL, 9% at 250 ng/mL and 16% at 500 ng/mL; i.e., caspase-3 activation was dose-dependent (Fig 3A). There was a 5-fold increase in the active caspase-3 level in response to 250 ng/mL AaGroEL compared to the negative control (p<0.05, Fig 3A).

To further investigate caspase activity in AaGroEL-mediated apoptosis, cells were incubated with a general caspase inhibitor (Z-VAD-FMK, 50 μM) for 1 h at 37°C before adding 250 ng/mL AaGroEL. There was a significant decrease in the rate of apoptotic T cells in the cultures with the caspase inhibitor. AaGroEL-mediated T-cell apoptosis decreased from 23% to 14% in Annexin V-positive cells and from 20% to 13% in Annexin V-positive/7AAD-positive T cells when the general caspase inhibitor was added to the cultures (p<0.005, Fig 3B). Together, these results suggest that caspase-3 is involved in AaGroEL-mediated T-cell apoptosis (Fig 3A and 3B).

An external or internal apoptotic pathway can be triggered by various stimulants. There is more than one extrinsic apoptotic pathway, including the Fas/Fas ligand (Fas/FasL) and TNF/TNF receptor (TNF/TNFR) pathways. These pathways induce apoptosis in certain cell types through the assembly of the death-inducing signaling complex (DISC) and subsequent caspase-8 activation. The initiator caspase-8 can directly activate the effector caspase-3, which causes cell apoptosis. Therefore, AaGroEL-treated cells at 48 h and 72 h were analyzed for the presence of caspase-8 by western blot detection. Caspase-8 is a proenzyme (55/50 kDa) that is proteolytically cleaved into smaller subunits of 40/36 (doublet) and 23 kDa upon receptor-ligand interaction. A procaspase-8 (55/50 kDa) band was identified in both AaGroEL-stimulated and RPMI-treated negative control cells (Fig 3C). However, cleaved bands of caspase-8, 40/36 kDa and 23 kDa bands, were identified only in the AaGroEL-stimulated cells (Fig 3C). These data indicated that caspase-3 and caspase-8 are involved in AaGroEL-mediated T-cell apoptosis (Fig 3A–3C).

**Fig 4:**
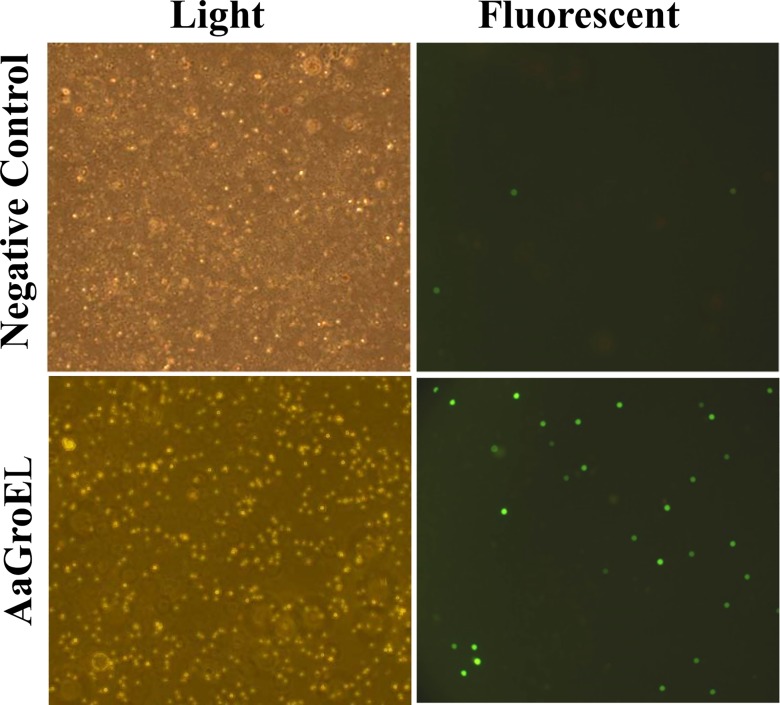
GroEL induces DNA fragmentation. PBMCs were treated with AaGroEL (250 ng) for 72 h. RPMI alone was used as a negative control. At the end of the incubation, the cells were stained using the MEBSTAIN apoptosis detection kit and analyzed by Olympus fluorescence microscopy (magnification x20). Cells with fragmented DNA were positively labeled with FITC-conjugated dUTP.

DNA fragmentation is the last stage of the apoptotic process. We showed that AaGroEL-mediated apoptosis induces caspase-3 activation. An active caspase-3 is the irreversible point of apoptosis and causes DNA fragmentation. Therefore, PBMCs were treated with AaGroEL for 72 h and subjected to the DNA fragmentation assay. Many FITC-conjugated dUTP-labeled cells were observed, indicating the presence of fragmented DNA in the AaGroEL-stimulated cells. It was also noted that there was an increase in the number of dUTP-FITC-positive cells in the AaGroEL-stimulated cells compared to the negative controls (Fig 4).

### The purification process does not contribute to GroEL-induced T-cell apoptosis

**Fig 5:**
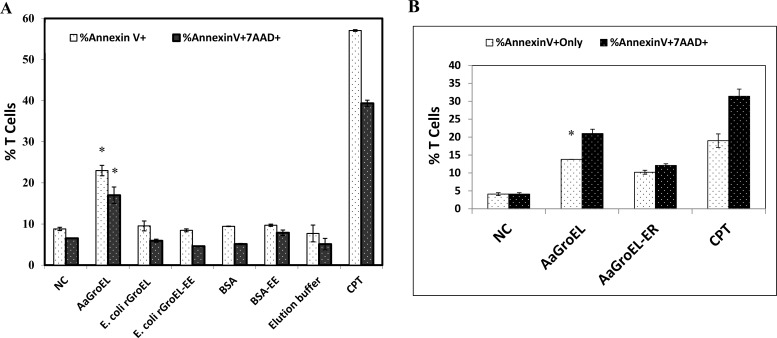
The purification process and LPS do not contribute to T-cell apoptosis. (A) Effect of purification on apoptosis. PBMCs were cultured for 48 h with endogenous electroeluted AaGroEL, *E*. *coli* rGroEL, electroeluted *E*. *coli* rGroEL, BSA and electroeluted BSA at 250 ng/mL. (B) Effect of LPS on apoptosis. Cells were cultured for 48 h with electroeluted, purified AaGroEL (AaGroEL) and LPS-removed AaGroEL (AaGroEL-ER) at 250 ng/mL. RPMI was used as a negative control, and camptothecin (CPT, 4 μM) was a positive control. Cultured cells were labeled with anti-CD3 antibody, Annexin V and 7AAD and analyzed by flow cytometry. Error bars represent the standard deviation, and * indicates p<0.05. The data are representative of 3 experiments with samples from different donors.

It is possible that the purification process may introduce contamination that compromises the antigenic properties of the protein, even if in trace amounts. Therefore, it is important to demonstrate that the purification process does not contribute to apoptosis. To address this concern, two controls were included in the study: commercially available *E*. *coli* rGroEL protein [16] and BSA, both of which were electroeluted concurrently with the AaGroEL protein. PBMCs were cultured with electroeluted-AaGroEL, electroeluted-*E*.*coli* recombinant GroEL (*E*.*coli*rGroEL-EE) or electroeluted-BSA (BSA-EE) for 48 h. BSA with or without electroelution had the same effect on T cell apoptosis, nearly the same as the negative control (Fig 5A). The electroeluted- *E*. *coli-* recombinant-GroEL protein exhibited no antigenic properties after purification. The elution buffer had no apoptotic effect compared to the RPMI culture medium. When PBMCs were cultured with electroeluted-AaGroEL, 23% of the cells were only Annexin V-positive T cells (p<0.05, Fig 5A). There was a statistically significant difference (4-fold difference) between the AaGroEL-treated cells and the negative control at 48 h (p<0.05, Fig 5A). These results clearly show that the GroEL-induced apoptosis is not due to anything associated with the purification process but is likely due to the antigenic properties of AaGroEL.

LPS contamination is an important issue in antigen preparation, and must be minimized. To evaluate the potential contribution of LPS to T-cell apoptosis, PBMCs were cultured in the presence of AaGroEL from which LPS had been removed prior to addition to the culture. Following LPS removal, endotoxin-depleted AaGroEL (AaGroEL-ER) still induced apoptosis in 10.2% of the T cells compared to 4.1% in the negative controls (p<0.005, Fig 5B). Camptothecin (CPT) was used as a positive control of the apoptosis. Altogether, these findings suggest that the apoptosis of T cells was not due to steps in the purification process or LPS contamination (Fig 5A and 5B).

## Discussion

Apoptosis, or programmed cell death, is one way bacteria invade or manipulate host cells [17]. The oral pathogen *A*. *actinomycetemcomitans* possesses many virulence factors, but unfortunately, the GroEL protein from this pathogen has not been extensively studied. In this study, we investigated the apoptosis-inducing effect of AaGroEL on human T cells. The data showed that the endogenous AaGroEL protein induces human T-cell apoptosis according to an alteration in cell size and plasma membrane structures and the activation of caspase-3 and caspase-8 and detected DNA fragmentation.

To use the GroEL protein as an antigen, ATP affinity chromatography-purified AaGroEL [15] was electroeluted and confirmed by western blot analysis (Fig 1A–1C) and mass spectrophotometry (data not shown). All the purification steps were tested for a potential contribution to T-cell apoptosis (Fig 5A). Furthermore, purified AaGroEL protein samples were tested for LPS contamination; Fig 5B indicates that AaGroEL-induced T-cell apoptosis was not due to any such contamination.

First, we showed that AaGroEL induces T-cell apoptosis by observing plasma membrane changes that were detected with the exposure of PS on the cell surface. The dose-response data revealed that the LD50 of the AaGroEL protein was 500 ng/mL, which induced apoptosis in approximately 50% of the T cells (Fig 2A). However, 250 ng/mL of protein was used in all subsequent experiments because this dose was sufficient to induce apoptosis (4-fold difference compared to the negative control, p<0.05, Fig 2A). Endogenous AaGroEL-induced apoptosis was dose-dependent (Fig 2A). By monitoring the plasma membrane changes, we also determined that endogenous AaGroEL induces apoptosis in a time-dependent manner (Fig 2B and 2C).

Active caspase-3 is a good indicator for monitoring the apoptotic process [13]. Therefore, active caspase-3 levels were also measured in AaGroEL-stimulated cultures. When different doses of AaGroEL were applied to cultures, there was a dose-dependent activation of caspase-3 (Fig 3A). Furthermore, the pretreatment of PBMCs with the general caspase inhibitor Z-VAD-FMK resulted in a decrease in the production rate of early- and late-apoptotic T cells (Fig 3B). This indicates that caspase activation is involved in T-cell apoptosis in response to AaGroEL (Fig 3B). In addition, cleaved 40/36-kDa and 23-kDa bands of caspase-8 were detected in AaGroEL-stimulated cells (Fig 3C). There was also increased DNA fragmentation in the AaGroEL-treated cells (Fig 4). Overall, the data demonstrated that endogenous AaGroEL protein induces T-cell apoptosis as assessed by plasma membrane changes, the activation of caspase-3 and caspase-8 and DNA fragmentation. The apoptotic activity of endogenous GroEL indicates that the AaGroEL protein is a virulence factor that induces T-cell apoptosis.

It is known that bacterial GroEL homologues from other pathogens can induce apoptosis [18, 19]. For instance, *in vitro*, the *Chlamydia trachomatis* Hsp60 induces apoptosis in primary human trophoblasts, placental fibroblasts and the JEG3 trophoblast cell line through toll-like receptor 4 (TLR4). In primary placental fibroblasts, chlamydial heat shock protein 60-induced apoptosis is caspase-dependent, whereas in JEG3 trophoblast cell lines, apoptosis is caspase-independent, suggesting a host cell-type-dependent apoptotic response [18]. Additionally, *Chlamydia pneumoniae* Hsp60 (cHsp60) is associated with caspase-dependent/independent apoptotic pathways in human atheromatous plaques of cHsp60-positive coronary artery disease patients [19]. Previously, it has been shown that AaGroEL is cytotoxic to epithelial cells. The long-term exposure of skin keratinocytes (the HaCaT cell line) to Hsp60 isolated from *Actinobacillus actinomycetemcomitans* increased the rate of epithelial cell death [9, 10, 20]. It has also been shown that AaGroEL partially inhibits stress-induced cell death in skin keratinocytes through the activation of ERK and the inhibition of caspase-3 [21].Additionally, AaGroEL favors the stimulation of CD8 T-cell apoptosis [22].

The present study showed through plasma membrane changes and caspase-3 and caspase-8 activation that the purified endogenous heat shock protein GroEL of *A*. *actinomycetemcomitans* induces apoptosis in primary human T cells. It is clear from these data that the GroEL protein of *A*. *actinomycetemcomitans* is among the apoptosis-inducing heat shock proteins. It is thus possible that AaGroEL can utilize apoptosis to manipulate the immune response. Further studies are required to investigate the molecular mechanisms of GroEL-mediated T-cell apoptosis.

## Abbreviations

Aa: *Aggregatibacter actinomycetemcomitans*
AaCE: *A*. *actinomycetemcomitans* sonic cell extract
BSA: bovine serum albumin
CDT: Cytolethal Distending Toxin
LTX: Leukotoxin
EE: electroeluted
Hsp: heat shock protein
Hsp: *A*. *actinomycetemcomitans* GroEL
LPS: lipopolysaccharide
AaGroEL-ER: LPS (endotoxin)-depleted AaGroEL
PBMCs: peripheral blood mononuclear cells
PS: phosphatidylserine
7AAD: 7-amino-actinomycin D
rAaGroEL: recombinant AaGroEL
